# Ten simple rules for creating a scientific web application

**DOI:** 10.1371/journal.pcbi.1009574

**Published:** 2021-12-09

**Authors:** Jessica L. Burnett, Renee Dale, Chung-Yi Hou, Gabriela Palomo-Munoz, Kaitlin Stack Whitney, Steve Aulenbach, Robert Sky Bristol, Denis Valle, Tristan P. Wellman

**Affiliations:** 1 U.S. Geological Survey, Core Science Systems Science Analytics and Synthesis, Lakewood, Colorado, United States of America; 2 Donald Danforth Plant Science Center, Olivette, Missouri, United States of America; 3 Apogee Engineering, Colorado Springs, Colorado, United States of America; 4 School of Natural Resources, University of Nebraska-Lincoln, Nebraska, United States of America; 5 Rochester Institute of Technology, Rochester, New York, United States of America; 6 School of Forest Resources and Conservation, University of Florida, Gainesville, Florida, United States of America; Carnegie Mellon University, UNITED STATES

## Abstract

The use of scientific web applications (SWApps) across biological and environmental sciences has grown exponentially over the past decade or so. Although quantitative evidence for such increased use in practice is scant, collectively, we have observed that these tools become more commonplace in teaching, outreach, and in science coproduction (e.g., as decision support tools). Despite the increased popularity of SWApps, researchers often receive little or no training in creating such tools. Although rolling out SWApps can be a relatively simple and quick process using modern, popular platforms like R shiny apps or Tableau dashboards, making them useful, usable, and sustainable is not. These 10 simple rules for creating a SWApp provide a foundation upon which researchers with little to no experience in web application design and development can consider, plan, and carry out SWApp projects.

## Introduction

In recent years, scientific web applications (SWApps) are more commonly observed in classrooms, for outreach, and in stakeholder engagement activities in the biological and environmental sciences. SWApps are increasingly used as decision support tools in the natural resources conservation and management communities [1], for teaching modeling and data analysis techniques [2], and are now a common tool used by researchers to apply new or complex analyses on their own data. SWApps can provide services that are complementary to the predominant forms of scientific communication (e.g., journal publications and technical reports), and, in some cases, have unique advantages over their more static counterparts. When thoughtfully designed, SWApps can (i) deliver information quickly, iteratively, and sometimes in real time; (ii) communicate science to audiences with diverse backgrounds and disciplinary expertise, as opposed to communicating with a narrow audience (e.g., peer-reviewed journal articles); and (iii) empower and engage end users to produce bespoke results targeted for a specific project or management decision (e.g., customized visualizations, analyses, and summarizations). Given the apparent rise in SWApp creation and use in these disciplines, and because most scientists receive little or no training in web design and development, an introductory guide to SWApp development is timely.

**Fig 1:**
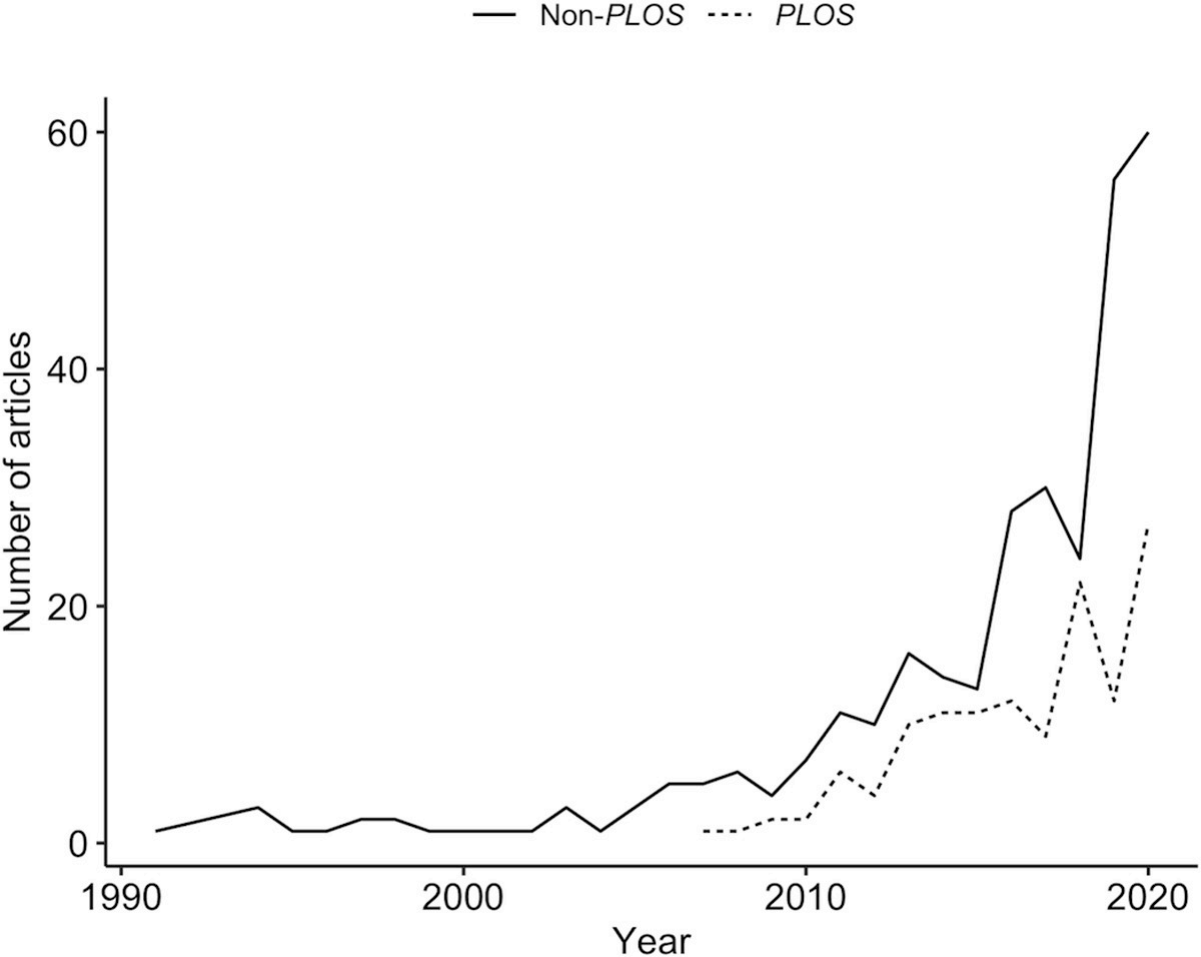
The number of publications (1991–2020) mentioning “web applications” (including Tableau, Shiny Apps, and WebGIS) across all PLOS publications (dashed line; PLOS) and in select publications in fields related to natural resources management (solid line; non-PLOS) has risen exponentially since approximately 2015. Data gathered from Web of Science on July 26, 2021 using the following advanced search Boolean: `TS = ("web app*" OR "shiny app*" OR "tableau" OR "webgis") AND (SU = ("Biodiversity & Conservation" OR "Environmental Sciences & Ecology" OR "Fisheries" OR "Forestry") OR SO = PLOS*)`.

The use of web applications to broadly communicate scientific understanding was highlighted during the first rise and peak of the ongoing Coronavirus Disease 2019 (COVID-19) pandemic [3,4]. In this context, SWApps were widely used to inform policy and decision makers [5], provide tools for contact tracing [6], and to actively communicate actual and probable COVID-19 case and death distributions across the globe [7]. SWApps are also used to facilitate decision-making to more narrow groups. By translating complex modeling techniques and results into digestible, interactive products or information, SWApps are also commonly used to facilitate decision-making processes in the natural resources management and policy fields. For example, they inform endangered species listing decisions under high uncertainty (e.g., [6]), facilitate biodiversity land management and planning processes [8], and are “relied on heavily” for assessing potential impacts of federally proposed or funded projects on critical habitat (personal communication with a user regarding [9]). As is suggested by the number of publications discussing or mentioning web applications in recent years (Fig 1), SWApps are becoming ubiquitous in scientific communication. However, without an efficient and effective means of creating and communicating information, even the most well-intentioned scientific products may be underutilized.

Many researchers and organizations who develop SWApps for communicating science may lack training in web, web application, and software development. Although SWApps can easily be created without formal training in these fields, even a superficial understanding of web app design and development can lead to more relevant, effective, and long-lived products. A basic understanding of SWApp development is even more important in decision-making contexts (e.g., fisheries management: https://www.habitat.noaa.gov/protection/efh/efhmapper/; clinical: [10]), where the front end (the client side or what the user interacts with) of a SWApp is equally, if not more, important than the back end (the server side or the programming for delivering the interface and underlying data, information, and services).

**Fig 2:**
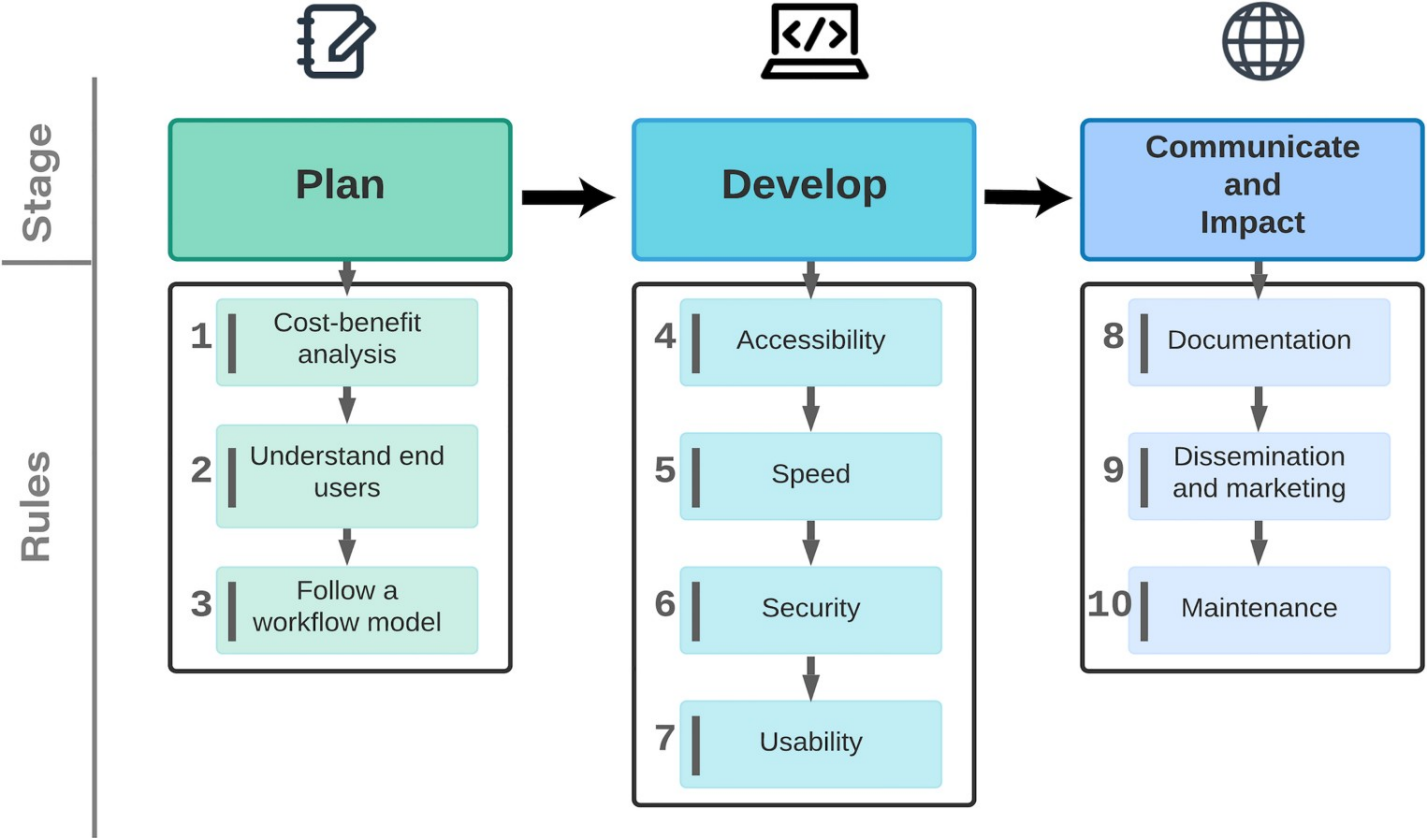
Overview of the 10 simple rules for creating a SWApp among 3 stages we have identified: planning, development, and communication. SWApp, scientific web application.

Here, we provide 10 rules for creating more accessible, sustainable, and useful SWApps. These rules (Fig 2) are largely adapted from recommended practices across the web and software development communities [1,3] and are provided in 3 stages: (i) Plan, (ii) Develop, and (iii) Communicate. In the planning stage (Rules 1 to 3), we emphasize the value of using project planning techniques, understanding end users, and conducting cost–benefit analyses. In the development stage (Rules 4 to 7), we focus on steps for improving the performance and sustainability of a SWApp. Finally, the communication stage (Rules 8 to 10) highlights key points of managing the communication life cycle of a SWApp, with a focus on 2-way interaction. These rules can be applied to projects of all sizes and budgets, but our solutions and recommendations are aimed to benefit individual researchers or small teams lacking formal training in web or web application development. We present the rules linearly but suggest readers consider them throughout a project’s life cycle. Implementing these rules will help improve the overall quality, impact, and sustainability of a SWApp.

## Stage I: Plan

### Rule 1: Understand the end user needs and define solution

Creating an effective SWApp benefits from understanding and accommodating the subject matter, perspectives, technical skills, and expectations of end users. We suggest 2 key points to consider before beginning development.

#### Define the service needed and outline the end product

Conducting basic market research (e.g., search the web and talk to end users) can help developers determine what, if any, solutions already exist for solving this problem [11,12]. Whenever possible, build off existing solutions to save time in the development stage. It may be necessary to further refine the project scope until the proposed solution is both a unique and valuable contribution and capitalizes on existing tools or technologies. Further, outline the minimum requirements that are needed to fulfill your end users’ needs and to successfully provide the service. These characteristics can be used as a template against which project completion can be compared, and additional services or features warrant consideration only when these minimum requirements have been met.

#### Understand end user characteristics

The user groups for a particular SWApp may have a wide range of disciplinary expertise, expectations, science needs, and technological experience. Combined with their motivations for using the SWApp, these characteristics will influence the way end users interact with your SWApp. If a project goal includes use of the SWApp by a broad or wide-ranging community, it is important to develop a SWApp that can simultaneously target multiple end user types. Incorporating the varying perspectives and needs of end users by “walking in their shoes” is a common methodology used in consumer products and web application projects [13,14]. A user-centered design emphasizes the importance of creating and evaluating the SWApp through the users’ perspectives [11]. Doing so requires at least a basic understanding who the end users are, their needs, and the reasons they will be interacting with the SWApp.

### Rule 2: Weigh the costs and benefits of the project

Platforms and templates exist for quickly prototyping web applications (e.g., Shiny apps, Spotfire, Dash, and Tableau), but making them effective, efficient, and trustworthy tools for scientists, decision makers, and other science users can take substantial time and resources. When considering a SWApp project, researchers should conduct a cost–benefit analysis to ensure that they are making the best use of their time and resources and that of their stakeholders or funders. Although more sophisticated methods exist, like scenario planning [15], most readers will benefit from the simplest technique: weighing the pros and cons. The pros and cons of each SWApp project will be unique with respect to the project objectives, disciplinary content, and team member expertise and expectations. To help determine whether to pursue a SWApp project, we suggest, however briefly, considering the costs and benefits along the following axes: knowledge transfer, money, and time (Table 1). Given that web apps are not yet ubiquitously rewarded in many professional scientific fields (e.g., academia and research organizations), it is particularly important for early career researchers to consider how the project and deliverables might affect their current and future career goals.

**Table 1:** Potential costs and benefits for researchers to consider when creating, participating in, or leading a SWApp project.

	Potential benefits (+)	Potential costs (−)
Knowledge transfer	- Enhances or accelerates future web application projects	- Unforeseen problems requiring unbudgeted time and resources
	- Delivering valuable products and services to a community	- Limited usage by the community minimizes impact of knowledge transfer
		- Web application can be misinterpreted or used in inappropriate ways
Monetary	- Can be used as a template for future SWApps	- Maintenance costs including personnel time, computing, and hosting may be prohibitive when poorly defined
	- Web application reduces operational costs compared to other forms of disseminating information	- Limited value to taxpayers when usage is narrow, or service is not timely or relevant
Time investment	- Learn new skills or technologies	- Limited organizational rewards or incentives
	- Diversifies professional portfolio	- Opportunity cost (time spent creating app distracts from research)
	- Research may show broad impact if addressing topics that have an influence in other areas outside of the intended field (e.g., controversial topics and epidemiology)	- Research contribution might face unexpected scrutiny if web application has broad impact or addresses a controversial topic
		- Difficult to demonstrate impact

SWApp, scientific web application.

### Rule 3: Follow a project workflow model

To design, create, and release products on time and on budget, most modern software and web development projects are guided by workflow models (also referred to as life cycles) [13,14,16]. Workflow models structure projects by outlining when and how components will be designed, created, deployed, and maintained. Although workflow models exist specifically for web application development projects, SWApp projects may benefit more so from software development workflow models for 2 reasons. First, many SWApps will incorporate technical components (e.g., statistical model and complex data visualization) that will benefit from aspects explicitly addressed in software workflow models (e.g., software testing and incremental software development). Second, web application workflow models disproportionately emphasize the user interface and user experience (UI/UX). In some cases (especially for prototypes), the UI/UX will be unimportant. Once the project objectives have been defined (Rule 1), identify and review relevant workflow models from among those most used in the software and web development communities (Table 2): AGILE, Iterative, Prototyping, and Spiral. Although they are among the most commonly used workflow models in software development, the Big Bang, DevOps, V-model, and Waterfall workflows are not discussed here given their limited relevance to most SWApp projects [13,17–19].

**Table 2:** A brief guide for identifying workflow models most appropriate for a SWApp project.

Project characteristic	AGILE	Iterative	Prototyping	Spiral
The project is a prototype or is not necessarily expected to end in a finished product	+		+	-
Project is large, complex, and/or high risk	-	+	-	+
The project can be broken into small, distinct, and noninterdependent deliverables (e.g., software requires many functions, UI requires many smaller features)	+	+		
Project or app has a long expected life span	-		-	+
Major changes are expected in requirements, timelines, or expectations over the project period	+	+	+	+
Early versions need to be functional and are available to end users throughout development	+			+
End user feedback will be sought early and often	+	+	+	+
The underlying information or technologies are expected to change		+	-	+
Documentation is lengthy and complex	-	+	-	
Software testing is important (app new, previously untested, or complicated software)	+		-	

Benefits and downsides to selecting a particular workflow model are indicated using the plus (+) and minus or hyphen (-) signs, respectively.

SWApp, scientific web application; UI, user interface.

AGILE is often used to describe workflow models that emphasize iterative processes; however, it can be considered as a single workflow model [19]. AGILE is most useful when end user or client satisfaction is a top priority and when a minimally functional product is expected very early in the project [20]. In this workflow, the planning, requirements, design, coding, and testing phases are iteratively cycled through, each cycle culminating in an updated, functional SWApp. In an Iterative workflow, the project cycles through the phases of design, development, testing and implementation in very small increments. Here, end user or client feedback is sought and incorporated early and often. Prototyping (or “rapid prototyping” per [1]) works best when the goal is to produce a minimally functional product quickly. Prototypes are often created to propose an idea or to generate or estimate interest in a service. In Prototyping, the UI and end UX are emphasized, whereas the logic or back end of the SWApp is likely to change after initial feedback. Although they may end in project termination, prototypes can serve as the foundation for future product(s). With the primary goal of minimizing risk, the Spiral workflow model iteratively cycles over 4 distinct activities (together, called a spiral): (i) identify requirements and objectives; (ii) design product; (iii) develop product; and (iv) evaluate, test, and plan. This model provides opportunity to incorporate end user feedback or lessons learned throughout the project life span and delivers a functional product at the end of each spiral.

These and other models have been developed and refined to guide projects of varying budget sizes, end user or stakeholder involvement, complexity, and expected product life span [13,14]. Use these workflow models, however loosely, to improve the efficiency and quality of the SWApp design, development, and testing phases and to help keep projects on target.

## Stage II: Develop

### Rule 4: Make it accessible (reduce the digital divide barrier)

Science communication cannot reach its full potential when it excludes any demographic [21,22]. To maximize the number of users and encompass community needs, SWApps should be broadly applicable for the spectrum of expected users. A SWApp is fully accessible only when it is available to and is usable by all the user base possessing different demographic characteristics and needs. This includes providing content in different languages when appropriate and addressing visual impairments such as color blindness. Designing and iteratively evaluating the SWApp for accessibility early and throughout the project ultimately saves time and resources. To maximize accessibility, we suggest evaluating SWApps along 3 axes: speed of operation, disability accessibility, and language barriers.

#### Consider speed of operation

An end user’s ability to interact with interactive web products like SWApps is limited by available bandwidth or the maximum download and upload rates. Accessibility to those with limited or slow internet access can be improved by optimizing loading times, using cloud computing for computationally expensive operations, and using caches to retrieve external data and information where possible. Limitations from computational demands can be reduced by using data caching [23], periodic preprocessing of common operations, and using cloud services that which provide resource-intensive computational capacities via remote virtual (remote) computing platforms. Further, many open-source (no licensing cost) web applications such as Python Dash or R Shiny can be deployed within browsers locally, such that no internet connection is required to use it after the initial download.

#### Ensure accessibility for people with disabilities

SWApps should be equally usable by those with sensory and physical disabilities [24,25]. Accessibility can be planned and accounted for throughout development, and it is critical to not assume the SWApp is accessible without explicitly testing it. Basic evaluations for digital accessibility can be done quickly using open-source tools, including browser extensions for colorblind friendliness, screen reader compatibility tests, and leveraging algorithms for estimating minimum reading levels of text-based content [26]. Examples of open-source tools for evaluating for disability accessibility include https://pa11y.org/, https://open-indy.github.io/Koa11y/, https://github.com/oftheheadland/Colorblindly, and https://readaloud.app.

#### Lower the language barrier

Language barriers are among the most common contributors to the digital divide, disproportionately affecting users who are from non-English language backgrounds [27]. Many modern web platforms provide language translation plugins but will likely require quality control by someone capable of effectively communicating science in that language. Whenever possible, we suggest incorporating multilingual components of the SWApp (e.g., via pop-ups or alternative versions), especially if the content and/or services provided can be used by decision makers who are not fluent in the SWApps default language.

### Rule 5: Optimize loading speed

Loading speed (sometimes referred to as performance) is an often overlooked but important aspect of SWApps that can negatively affect usability and perceived quality. Speed is affected primarily by the number of connections or number of users, software technology type, volume of data, and the overall complexity of the SWApp. To identify and resolve issues early, we recommend evaluating SWApps for speed early and often; however, speed solutions that improve one component may slow down other components. Therefore, it is important to compare speed before and after implementing solutions. Although more advanced technical solutions exist for optimizing speed, here, we suggest 4 activities that can be implemented by novice developers.

#### Evaluate speed

First, use an online website speed test tool to evaluate and identify the exact locations of and wait times for app components. Some website speed tools also identify other important issues (e.g., 404 pages, empty content) and provide general suggestions for resolving issues identified (e.g., https://tools.pingdom.com). Advanced scientific programmers can also use benchmarks [24] to identify computational bottlenecks. If long wait times are unavoidable or are difficult to ameliorate, inform the user of the wait times using progress bars or messages [25].

#### Cache objects and precomputed tasks

Caching is the process of storing previously created objects, like data queries or model results, such that a request grabs the stored object rather than performing the entire task again. Caching is used to enhance load speeds for websites but can also be applied to computational tasks. SWApps with speeds inhibited by complex models or lengthy data processing tasks may benefit from precomputed caches, but only if cached objects are not relatively large. If necessary, we recommend caching the most retrieved information or queries or other objects that are central to most bespoke queries or used services (e.g., a commonly used remote dataset for a custom model).

#### Compress images and graphics

Often, images account for a disproportionate amount of web application sizes. Unless image quality is of the utmost importance to a SWApp, all images and graphics can be formatted and compressed. Consider removing unnecessary images, graphics, and animations to improve load times.

#### Rightsize your hosting infrastructure

Where and how to host a SWApp involves trade-offs relative to the application type, level of institutional resources, and associated costs. Before committing to any host, use online server speed checkers to evaluate your options (e.g., https://www.bitcatcha.com/). Some hosting solutions may even help reach targeted audiences (examples of free hosting solutions geared toward scientific research dissemination include https://www.cyverse.org/ and https://scigap.org/). Although most cloud-based solutions offer low- or no-cost options for getting websites up and running quickly, they may have performance limitations. Virtual private and dedicated servers are more expensive than cloud-based solutions but may be appropriate for computationally expensive apps or for those with high expected usage (e.g., >500 hours of monthly active use).

### Rule 6: Ensure security

Basic security flaws can contribute to distrust in a web application, which can influence a loss of existing and potential users. Although more advanced security solutions exist, SWApps should have a minimum of 2 security measures: encrypted connections and ethical and legal user data collection.

#### Always use encrypted connections

Web applications should always be hosted using a secure (https://, encrypted) protocol instead of a nonsecure (http://, non-encrypted) protocol [28]. A user visiting a nonsecure website will likely be warned and may either choose to not load the web page or may be entirely blocked by browser, antivirus, or network policy checks. Some recent SWApps have not implemented this basic security feature.

#### Declare user data collection

Collecting data on how your SWApp is being used, and in some cases by whom, can help identify potential areas of improvement. These data can also be used to demonstrate value to superiors and funders and help determine next steps. Any amount of user or usage data collection should be explicitly and immediately declared to the user (e.g., pop-up window upon initial load, terms, and conditions be reviewable later). Unless necessary, do not collect personally identifiable information. Finally, ensure the SWApp is in compliance with national, regional, and organizational policies on user data collection (e.g., EU General Data Protection Regulation).

### Rule 7: Maximize usability

Usability refers to the ease with which users can identify and accomplish tasks within a SWApp [29,30]. Usability is typically evaluated for 5 characteristics: efficiency, learnability, memorability, errors, and satisfaction [31]. Poor usability can deter a user from continuing to use the SWApp or recommending to others. While there are many techniques and strategies that can be used to design and improve usability, here, we highlight 2 basic ways that researchers can evaluate usability of a SWApp: self-evaluation and working with end users.

#### Self-evaluate the elements for functionality, consistency, and intuitiveness

Although it may seem obvious, an important step in SWApp development is ensuring all components of the SWApp work as expected. We recommend researchers use both automated testing services (e.g., https://web.dev and https://powermapper.com) and self-evaluation techniques to evaluate the functionality, consistency, and intuitiveness of the SWApp. First, evaluate all elements for functionality by ensuring that each element (e.g., image, hyperlink, and radio button) works as expected. For example, all hyperlinks point to the correct place, all images should load properly and have appropriate aspect ratios, and analytical results should be presented in the correct format. Next, evaluate a specific element type (e.g., hyperlinks, text, images, and videos) or group of elements (e.g., images and figures or hyperlinks and text) for consistency. Elements are consistent when each instance has similar visual characteristics (e.g., underlined text with unique color always represents a URL and header and body texts are distinct) and/or performs the same action (e.g., clicking a radio button results in a selection from a list). Finally, ensure that all icons, hyperlinks, radio buttons, dropdown lists, and pop-ups are immediately recognizable, or intuitive, to the visually abled [32]. Although minor solutions, fixing these issues can improve the overall UX and consequently a willingness to reuse the SWApp.

#### Work with end users to improve the UX

Key objectives of professional usability testing are to understand how users interact with a web application and interpret the available services and to gauge the overall UX [33]. A simple but informative form of usability testing can be achieved by directly observing how a nonaffiliated, volunteer end user (preferred), colleague, or member of your own team (least preferred) interacts with the web application [34]. In this scenario, the volunteer will use the web application (preferably for the first time) under your observation. The volunteer is asked to share their running thoughts such that the observer can understand points of confusion, frustration, or satisfaction with the web application. Depending on the services provided, you can either request the volunteer to perform a particular task (e.g., answer a research question), obtain a certain output, or can allow them to freely explore the web application without direct supervision [35,36]. Include 2 of the many examples providing basic introductions, templates, and resources for usability testing. The insights gained from usability testing can then be used to improve both the visible and nonvisible features within a SWApp.

## Stage III: Communicate

### Rule 8: Provide documentation

SWApp documentation encompasses the suite of written or otherwise recorded knowledge associated with developing or using the SWApp interface and its underlying science and software. We briefly review the benefits of SWApp documentation as created for 2 purposes: for the end users and for other researchers, developers, and citers.

#### Documentation for informing and guiding end users

Providing relevant, digestible, and easy-to-find information is a key method for communicating the purpose, value, and potential uses of a SWApp. End user documentation should be catered appropriately to the technical or subject matter expertise of the audience and should reflect end user needs (see Rule 7). Common end user documentation for SWApps include in-app communications (e.g., pop-ups), links to external resources like articles or user manuals, text descriptions, and audio or video walk-throughs. These can include generalized information, such as an overview of the app services and features, or can include specialized information, such as case study examples catered to a specific end user audience.

#### Technical documentation for citers, researchers, and developers

Technical documentation can serve as the reproducibility backbone of SWApps, but also provides key information for others wishing to credit your work. Minimum elements of technical documentation include any relevant citation information [37], software licensing [38], computing environment information [39], and source code (or instructions) for reproducing the interface (e.g., the R scripts for Shiny Apps) and the underlying software (e.g., parameterizing models). Documenting the code base can help when revisiting the SWApp in the future and helps other researchers who wish to build upon the original source code [40]. Documentation that captures the underlying science not only helps with reproducibility, but also improves credibility of the SWApp itself and its underlying science.

### Rule 9: Get the SWApp noticed

SWApps provide a unique and valuable opportunity to communicate science to a diverse audience. Given their underrated value, few widespread options exist for effectively marketing SWApps.

#### Market to relevant audiences

Marketing methods can be dictated by the intended audience of a SWApp. If end users are largely in the academic sector, consider publishing in a relevant journal or promoting via related meetings/conferences. If the audience extends beyond academics, reach out directly to individual organizations, professional societies, and individuals in the community. Some organizations or communities of practice may have a listserv or collection of web pages within which you can market your app. Regardless of the end user audience, adopting the standard outreach and engagement technique of working closely with and appeasing the early adopters, who are often valuable for spreading the word, can be beneficial.

#### Leverage search engine optimization (SEO)

One of the easiest methods to improve discoverability of a SWApp is to leverage search engine optimization (SEO). SEO draws on metadata (e.g., site title, tagline, description, and keywords), load times (see Rule 5), security (see Rule 6), and frequency of updates of web pages to rank end user query results [41]. The most basic (and free) way to improve SEO ranking is to write metadata from the end user’s perspective. For example, if the target audience of a SWApp that generates animated maps of bird distributions is the general public, technical terms describing the scientific aspects (e.g., generalized additive modeling and spatial ecology) may not be useful. In other words, use words that the target audience would use in a search engine [41]. Keywords can be provided within the web application itself, the host web site or page, and in associated documentation such as source code repositories, user manuals, or manuscripts. Notably, improving the accessibility of a web page (discussed in Rule 4) can also help improve overall SEO rankings [42].

#### Understand limitations to findability and discoverability for SWApp platforms

It is important to recognize that some modern web application hosting platforms commonly used by scientists may prevent search engines from mining its web pages, which can negatively affect the chance of a SWApp appearing because of a search engine query. For example, the Shiny applications hosted at https://shiny.io are not indexed and therefore do not appear in search engine queries. Creating a static web page that links directly to the SWApp and contains relevant keywords will create metadata search-engine indexing, consequently improving chances of potential users discovering the SWApp in a web search.

### Rule 10: Periodically evaluate and update the app

SWApps should evolve, albeit discreetly, with ever changing technologies, user expectations, and the best available science. A SWApp should be evaluated at regular intervals to achieve 3 things: (i) understand usage; (ii) ensure usefulness (timeliness and relevance); and (iii) identify maintenance needs. We provide a series of steps for improving the quality of a SWApp after its release.

#### Evaluate usage

Web analytics (also referred to as usage statistics) is a series of methods for passively tracking whether a web page is being used. Basic analytics gained for SWApps include length of visit, return rate, and from what domain they accessed the app. If the SWApp is not being used or is not performing as expected by the end user (see Rule 7), there may be a need to refine or discontinue the app. Direct feedback from end users can help determine what components or services should be adjusted or added to meet their needs and expectations, whereas usage statistics can influence the decision to maintain or retire the app.

#### Maintain the app

Web application maintenance benefits from both timely responses to user-reported issues or feedback and from periodic check-ins and monitoring. Providing contact information or in-app feedback form facilitates quick response of unforeseen issues and bugs, while regularly scheduled reviews of the app can help identify minor or potential bugs and areas for additional development. The most common errors and bugs in SWApps include incompatibility errors (e.g., updates of third-party software), changes in hyperlinks or data sources, and errors after adding new or updating existing functionality. These errors can be identified quickly by regularly monitoring and scheduling recurring maintenance activities.

## Conclusions

SWApps provide a powerful outlet for communicating science to diverse audiences. The 10 simple rules for creating a SWApp (Fig 2) were adapted from the key concepts and recommended practices in the web and software development communities. Understanding the basics of web application planning (Stage I), development (Stage II), and communication (Stage III) may lead to more efficient use of researchers’ and developers’ time and, when applicable, use of stakeholder or taxpayer funding.

Our rules are meant to provide a brief introduction to creating more useful, usable, and sustainable SWApps, but only touch the surface of web application design and development. In addition to the topics and resources provided here, readers may benefit from further education in a few topics not addressed here: the fundamentals of web architecture (the components of and processes which comprise the World Wide Web); basics of front-end development (HTML, CSS, and JavaScript); basics of back-end development (e.g., web servers and application programming interfaces (APIs)). Free learning resources for these and other topics include the W3C (https://www.w3.org and https://www.w3schools.org) and Coursera (https://www.coursera.org).
